# Suicide ideation and subsequent self-harm: Variations in presentations, care and management during the Covid-19 pandemic

**DOI:** 10.23889/ijpds.v8i2.2331

**Published:** 2023-09-14

**Authors:** Siobhán Murphy, Dermot O'Reilly, Aideen Maguire, Emma Ross

**Affiliations:** 1 Queen's University Belfast, Belfast, United Kingdom

## Objectives

The COVID-19 pandemic and associated societal changes including access to emergency departments (ED) may have influenced suicide ideation (SI) incidence and the case fatality rates. This study quantifies the numbers of individuals presenting to ED with SI before and during the pandemic and examines risk of subsequent presentation to ED with self-harm and mortality.

## Methods

The Northern Ireland Self-Harm (SH) Registry provided data on suicide ideation and self-harm presentations across 12 ED departments in NI between January 2016 and September 2021. Linkage to health and mortality records provided follow up to September 2021. Cox proportional hazards regression models were employed to assess subsequent self-harm and mortality risk following initial presentation to ED with suicidal ideation.

## Results

Preliminary findings indicate that during the study period there were 21,601 ED presentations with SI between made by 9184 individuals of whom 2,011 subsequently went on to self-harm. Presentations with SI increased in the four years prior to the pandemic with the highest occurring in 2019. The number of individuals presenting to ED then decreased substantially in March/April 2020 leading to a 37% and 47% reduction, corresponding to the first “lockdown”. Presentations remained lower until June 2020 when restrictions started to ease. Compared to the pre-pandemic period, individuals presenting with SI during the pandemic were approximately 60% less likely to ED with subsequent self-harm (ORadj = 0.41, 95% CI 0.29, 0.59) even after adjustment for variations in the demographic profile of individuals, and the care and management they received at the ED.

## Conclusion

Rates of ideation followed similar trends to previous years except in the early months of the pandemic. Those presenting with SI during the pandemic conferred a reduced likelihood of re-attending at ED with subsequent self-harm compared to the pre-pandemic period. The possible underlying mechanisms behind these findings will be discussed.

